# Associations between pedestrian fatalities and air temperature, United States, 2001-2021

**DOI:** 10.5249/jivr.v17i1.1939

**Published:** 2025-01

**Authors:** David C. Schwebel

**Affiliations:** ^*a*^ Department of Psychology, University of Alabama at Birmingham, Alabama, United States.

**Keywords:** Pedestrian fatality, Traffic safety, Injury prevention, Climate change, Global warming

## Abstract

**Background:**

Pedestrian injuries killed almost 9000 Americans in 2021. Pedestrian fatality rates have increased annually since 2009. Many factors are associated with pedestrian fatalities, but one poorly-understood risk is the role of climate. We examined two primary sets of associations, those between pedestrian fatality crude rate (deaths per 100,000 population) and air temperatures in each US state (2012-2021) and those between national air temperatures and pedestrian fatality crude rate in each year from 2001-2021.

**Methods:**

Data were obtained from public-facing CDC WISQARSTM and NOAA Climate-at-a-Glance websites. Descriptive and correlational analyses were conducted.

**Results:**

Average air temperatures in individual US states correlated with pedestrian fatality crude rates in those states, r = 0.66. National average annual temperatures correlated with annual pedestrian fatality crude rates each year, r = 0.61. Secondary analyses considering correlations between annual pedestrian fatality crude rates and heating degree days, cooling degree days, and grid days over 70° F replicated primary results. Secondary analyses considering change in temperature from the previous year and pedestrian fatality crude rates were null.

**Conclusions:**

US pedestrian fatality crude rates are associated with air temperatures. Causality cannot be assumed, but possible explanations for the association include increased exposure to traffic during daytime walking in cooler climates and increased exposure to risk through more walking at night in warmer climates. If findings were translated into NOAA-estimated climate change scenarios of 1° of temperature change over 30 years, correlational associations from the past two decades maintained unchanged, and population presumed stable, between 430-755 additional pedestrian fatalities could occur annually in the future.

## Introduction

Pedestrian injuries killed almost 9000 Americans in 2021, a figure that has increased annually since 2009. Experts attribute the magnitude of pedestrian fatalities to a range of road engineering, pedestrian behavior, and driver behavior factors.^1^ Experts attribute the rising rates to a wide range of factors also, including increasing frequency of distracted driving and distracted pedestrian behavior, successful public health efforts to increase walking and jogging for exercise, and increased numbers of SUVs and trucks on the roadways, which are more fatal in crashes than sedans.^2^

One risk factor has received only minimal attention, the role of climate on pedestrian fatality risk. A handful of publications suggest pedestrian activity, crashes and fatalities may be more common in warmer weather.^3-7^ People may tend to walk and run more in warmer environments, which leads to increases in pedestrian fatality exposure risk.^3^ Other publications report links between warmer temperature and broad road traffic injuries^8^ as well as warmer temperature and higher intentional injury rates,^9^ occupational injury rates,^10^ general injuries,^11^ and pediatric trauma admissions.^12^

Data from the National Oceanic and Atmospheric Administration (NOAA) in the United States (US) indicate a 0.32° F increase in global temperature each decade since 1981.^13^ Is an increase in pedestrian fatality rates possibly related to climate change? We considered two associations between air temperature and pedestrian fatality rates: (a) temperatures in each US state and the pedestrian fatality crude rate (deaths per 100,000 population) in that state over the most recent decade of available data, 2012-2021; and (b) nationwide US temperatures annually between 2001 and 2021, and pedestrian fatality crude rate in that year. We reasoned that strong associations in both evaluations would suggest possible links between air temperature and pedestrian fatality rates.

## Methods 

Data were obtained from publicly-accessible sources. Pedestrian fatality crude rates (deaths per 100,000 individuals) were obtained from the US Centers for Disease Control and Prevention (CDC) WISQARSTM website (https://www.cdc.gov/injury/wisqars/index.html). The CDC considers these data to be authoritative and accurate for use in both research and public health decision making. Temperature data for 49 US states (excepting Hawaii, which are unavailable) were obtained from the US National Oceanic and Atmospheric Administration’s (NOAA) National Centers for Environmental Information Climate at a Glance website (https://www.ncei.noaa.gov/access/monitoring/climate-at-a-glance/). NOAA considers these data appropriate for research on climate variability.

Following online download, data were compiled and primary hypotheses were analyzed descriptively and through two Pearson correlations: (a) average temperature and pedestrian fatality crude rate between 2012 and 2021, with state as the unit, and (b) average national temperature between 2001 and 2021 and pedestrian fatality crude rate, with year as the unit.

We also conducted three secondary analyses. First, we conducted a sensitivity analysis with two alternative measures of average temperature, heating degree days (HDDs) and cooling degree days (CDDs). HDDs and CDDs offer a metric calibrated around temperatures above or below 65° F (18° C). Based loosely on the extent to which homeowners might turn on their home’s heating or air-conditioning units, respectively, each degree of daily average temperature above 65° F equates to a like amount of CDDs, and each degree below 65° F equates to a like amount of HDDs.

Second, we recognized that very hot temperatures may lead to reduced pedestrian activity. We therefore computed a newly-developed metric, grid days over 70° F (21° C), to tally the number of days per year when grid cells in the country (defined in line with the 0.5° latitude by 0.5° longitude resolution of NOAA's Climate Prediction Center (CPC) gridded temperature dataset, with 50ºN defining the northern boundary, 25ºN defining the southern boundary, and adjustments made to incorporate southern New Mexico) rose above 70° F but below 90° F (32° C). This metric offered a test of associations between pedestrian fatalities in a warm but comfortable air environment, excluding days when the temperature exceeded 90° F. 

Data for HDDs, CDDs, and grid days over 70° F were derived and processed using the NOAA/CPC Daily Global Unified Temperature dataset (https://psl.noaa.gov/data/gridded/data.cpc.globaltemp.html). They excluded Alaska and Hawaii due to data availability.

Last, we considered associations between recent change in air temperature and pedestrian fatalities rather than associations between current temperature and pedestrian fatalities. With climate change creating increasing temperatures, we reasoned that change year over year might be associated with pedestrian fatalities and therefore correlated the average national temperature change from one year to the next with the pedestrian fatality crude rate in the second year.

The study protocol was reviewed and received a non–human subjects research determination by the Institutional Review Board at the University of Alabama at Birmingham (IRB-300012170).

## Results

**Figure 1:**
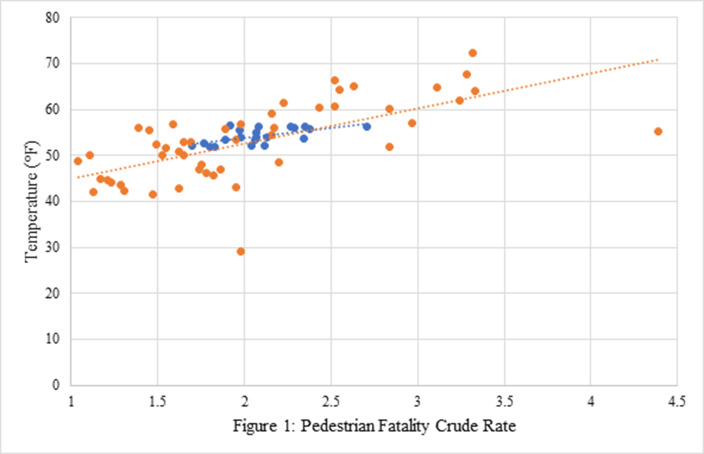
Orange points and trend line represent the pedestrian fatality crude rate (deaths per 100,000 population) and average temperature between 2012 and 2021, by state. Blue points and trend line represent the pedestrian fatality crude rate and average annual US temperature between 2001 and 2021, by year.

The average Fahrenheit temperature in individual US states between 2012 and 2021 ranged from 29.02 (Alaska) to 72.38 (Florida), with a mean of 53.00 and standard deviation (SD) of 8.49. The pedestrian fatality crude rate in US states over that time period ranged from 1.04 (Iowa) to 4.39 (New Mexico), with a mean of 2.04 and SD of 0.72. As shown in Figure 1, the two variables correlated strongly, r(47) = 0.66.

The average annual Fahrenheit temperature across the US between 2001 and 2021 ranged from 51.80 in 2011 to 56.60 in 2012, with a mean of 54.20 and SD of 1.74. The annual pedestrian fatality crude rate in the US over that time period ranged from 1.70 (2009) to 2.71 (2021), with a mean of 2.08 and SD of 0.24. As shown in Figure 1, the two variables correlated strongly, r(19) = 0.61. 

We conducted three sets of secondary analyses. First, sensitivity analyses considered correlations between the pedestrian crude rate in the US from 2001 and 2021 and HDDs and CDDs. On average during that timespan, HDDs decreased annually at a rate of 138 per year and CDDs increased at a rate of 343 per year. Pedestrian fatality crude rate correlated with annual HDDs and CDDs strongly, r(46) = -0.57 and 0.41, respectively.

Second, we considered associations between pedestrian crude rate in the US from 2001 to 2021 and grid days over 70° F each year. The number of grid days over 70° F decreased by an average annual rate of 240. Pedestrian fatality crude rate and grid days over 70° F correlated, r(46) = 0.31.

Last, we considered correlations between annual change in air temperature, computed as change from the previous year, and pedestrian crude rates in the US from 2002 to 2021. Annual change in temperature had a mean of .13° (standard deviation = 2.03, range from -3.3° from 2012-2013 to +4.8° from 2011 to 2012). The correlation was not significant, r(18) = 0.04.

## Discussion

Primary results suggest a strong association between air temperature and pedestrian fatality crude rates in the US, both by time (year by year) and place (state by state). We cannot assume causality in the associations, but if the current findings were translated into a climate change scenario of 1° of temperature change over 30 years, consistent with NOAA estimates over the past 40 years,^13^ then our first analysis would suggest a crude rate increase of 0.13 occurs with each 1° upward change in temperature by location. Given the current US population of 331,900,000 and assuming stable population levels, a crude rate increase of 0.13 equates to 430 additional annual US pedestrian fatalities. Using findings from the second analysis, we estimate a crude rate increase of 0.23 for each 1° of change in temperature by year. That figure equates to 755 additional annual pedestrian fatalities.

Our secondary results support primary findings. HDDs and CDDs were correlated with pedestrian fatality rates. The association between grid days over 70° F and pedestrian fatality rates explored whether very hot air temperatures – over 90° F – might lead to lower pedestrian fatality rates. The association between grid days over 70° F and pedestrian fatality crude rates was positive, but at a lower magnitude (effect size) than the other correlational analyses. This finding offers some indication that pedestrian fatality rates may not decrease with very hot air temperatures. Finally, our analysis of associations between change in air temperature over the past year and pedestrian fatality rates was null, suggesting current air temperature is more relevant to pedestrian fatality risk than recent annual changes in air temperature.

Why might hotter temperatures be associated with increased pedestrian fatalities? The most logical answer is a mediation path through increased exposure: more people walk when the temperature is more comfortable.^3^ Traffic volume also changes with temperature change; there is evidence that both vehicle^14-16^ and pedestrian^3,17^ volume decrease in cold weather.

Additional mediating, moderating, and third-variable factors may also contribute to the associations we report. When temperatures increase from warm to extremely warm, such as the case in large cities like Phoenix or Miami, pedestrians may choose to walk at night, presenting additional risk due to visibility and alcohol use.^18-19^ Temperature increases may also lead to increased exposure among vulnerable populations, including homeless individuals, a population that has grown over time, is more likely to live in warmer climates, and has elevated pedestrian fatality risk.^20-21^

Our study has limitations. Pedestrian fatalities are relatively rare, creating a low base rate for analytic purposes. Thus, we conducted analyses by larger periods of time and across larger geographic regions instead of dividing results into individual months or seasons, or considering regions within larger states. Future research should consider ways to examine change more granularly, looking within specific geographic areas that may have varying climate effects. One way to accomplish this goal may be to examine pedestrian injuries rather than pedestrian fatalities, as injuries are far more common than fatalities. Relatedly, we focused on air temperature but acknowledge that other climate conditions, including precipitation, icy/snowy conditions and very hot weather each may play a role in pedestrian fatality risk.^4,7^

Our study was also limited by including analyses of temperature by location as well as temperature by year. Our correlation between temperatures in each state and the pedestrian fatality rate in that state was an evaluation of whether pedestrian fatalities increase in warmer places, not in warmer years. It offers evidence relevant to our thesis that increasing temperatures through climate change may create increasing pedestrian fatality rates, but the link is indirect. Finally, we did not examine possible mediating and moderating effects that could impact the association between air temperature and pedestrian fatalities. Future research might incorporate variables such as homelessness rates, pedestrian activity rates, alcohol consumption rates, and other possible mediators and moderators to consider causal pathways that could explain our reported associations between pedestrian fatalities and air temperature over both space and time.

## Conclusion

The evidence for climate change is compelling,^13^ and the implications for public health are broad.^22^ These results suggest one more possible effect of climate change on human health: an increased risk of pedestrian injury, potentially due to the confluence of multiple factors associated with increased exposure of pedestrians to traffic during warmer weather. There may be particular risk among vulnerable individuals, including the homeless population.

We do not recommend efforts to decrease pedestrian behavior. To the contrary, pedestrian activity is both health-promoting and consistent with goals for environmental sustainability. Instead, we urge continued effort to consider our built environment and roadway infrastructure, assuring they provide safe options for pedestrian travel. Pedestrians can and must safely share our transportation infrastructure.

**Acknowledgment: **Thanks to Howard J. Diamond, Ph.D., of the Air Resources Laboratory, National Oceanic and Atmospheric Administration, for providing data used in this analysis and developing the idea of the grid days variable. Dr. Schwebel received no external financial support for the research, authorship, and/or publication of this article.
